# Alcohol and Other Substance Use Before and During the COVID-19 Pandemic Among High School Students — Youth Risk Behavior Survey, United States, 2021

**DOI:** 10.15585/mmwr.su7201a10

**Published:** 2023-04-28

**Authors:** Brooke E. Hoots, Jingjing Li, Marci Feldman Hertz, Marissa B. Esser, Adriana Rico, Evelyn Y. Zavala, Christopher M. Jones

**Affiliations:** ^1^Division of Overdose Prevention, National Center for Injury Prevention and Control, CDC; ^2^Division of Adolescent and School Health, National Center for HIV, Viral Hepatitis, STD, and TB Prevention, CDC; ^3^Division of Population Health, National Center for Chronic Disease Prevention and Health Promotion, CDC; ^4^Office of the Director, National Center for Injury Prevention and Control, CDC

## Abstract

Adolescence is a critical phase of development and is frequently a period of initiating and engaging in risky behaviors, including alcohol and other substance use. The COVID-19 pandemic and associated stressors might have affected adolescent involvement in these behaviors. To examine substance use patterns and understand how substance use among high school students changed before and during the COVID-19 pandemic, CDC analyzed data from the nationally representative Youth Risk Behavior Survey. This report presents estimated prevalences among high school students of current (i.e., previous 30 days) alcohol use, marijuana use, binge drinking, and prescription opioid misuse and lifetime alcohol, marijuana, synthetic marijuana, inhalants, ecstasy, cocaine, methamphetamine, heroin, and injection drug use and prescription opioid misuse. Trends during 2009–2021 were assessed using logistic regression and joinpoint regression analyses. Changes in substance use from 2019 to 2021 were assessed using prevalence differences and prevalence ratios, stratified by demographic characteristics. Prevalence of substance use measures by sexual identity and current co-occurring substance use were estimated using 2021 data. Substance use prevalence declined during 2009–2021. From 2019 to 2021, the prevalence of current alcohol use, marijuana use, and binge drinking and lifetime use of alcohol, marijuana, and cocaine and prescription opioid misuse decreased; lifetime inhalant use increased. In 2021, substance use varied by sex, race and ethnicity, and sexual identity. Approximately one third of students (29%) reported current use of alcohol or marijuana or prescription opioid misuse; among those reporting current substance use, approximately 34% used two or more substances. Widespread implementation of tailored evidence-based policies, programs, and practices likely to reduce risk factors for adolescent substance use and promote protective factors might further decrease substance use among U.S. high school students and is urgently needed in the context of the changing marketplaces for alcohol beverage products and other drugs (e.g., release of high-alcohol beverage products and increased availability of counterfeit pills containing fentanyl).

## Introduction

Adolescence is a critical phase of physical, cognitive, social, and emotional development and is frequently a period of initiating and engaging in risky behaviors, including alcohol and other substance use. The majority of adolescents engage in some form of substance use before finishing high school (*1*,*2*). Substance use during adolescence is associated with adverse health outcomes, such as mental health problems, teen pregnancy, and sexually transmitted diseases as well as consequences, such as delinquency, violence, and academic underachievement (*2*,*3*). Substance use initiation during adolescence can increase the risk for substance use later in adulthood and increase the risk for substance use disorders (https://addiction.surgeongeneral.gov/sites/default/files/surgeon-generals-report.pdf). Adolescent substance use is of particular concern as overdose deaths among adolescents have increased dramatically (*4*). The Drug Enforcement Administration has warned of readily available counterfeit pills containing highly lethal substances (e.g., illicit fentanyl) and other synthetic opioids that are designed to look like commonly misused prescription medications that might be contributing to these increases (https://www.dea.gov/press-releases/2021/05/21/dea-issues-warning-over-counterfeit-pills; https://www.dea.gov/sites/default/files/2021-05/Counterfeit%20Pills%20fact%20SHEET-5-13-21-FINAL.pdf). The alcohol industry and regulatory environment is also changing, including the release of high-alcohol content products (https://www.samhsa.gov/resource/ebp/implementing-community-level-policies-prevent-alcohol-misuse). In addition, alcohol-related deaths, including those involving other substances, have increased among adolescents (*5*).

In 2021, CDC’s Adolescent Behaviors and Experiences Survey (ABES) found that students experienced adversities and challenges during the COVID-19 pandemic, such as poor mental health, persistent feelings of sadness or hopelessness, suicidal ideation, and physical and emotional abuse, all of which are risk factors for substance use (https://www.cdc.gov/healthyyouth/data/abes/reports.htm). In addition, measures to protect adolescents from COVID-19 infection, such as remote schooling, social isolation, and event cancelation, might have contributed additional risk for adolescent substance use. One third of students participating in ABES who had ever drunk alcohol or used drugs used those substances more during the pandemic (*6*).

Other studies examining adolescent substance use during the pandemic have had varying findings. For example, the Monitoring the Future survey indicated declines in current marijuana use, alcohol use, and binge drinking when comparing 2020 and 2021 prevalence estimates (*1*). However, another study comparing prevalence estimates from the early stages of the pandemic to prepandemic estimates found increases in the frequency of both marijuana and alcohol use (*3*), and another demonstrated no change in the use of either substance (*7*).

The variability in the previous studies highlighted the need for additional studies of nationally representative data to assess changes in alcohol and other substance use before and during the pandemic. This report used Youth Risk Behavior Survey (YRBS) data to improve understanding of how substance use changed before and during the COVID-19 pandemic. Specifically, this report examined overall trends in alcohol and other substance use, characterized changes in alcohol and other substance use by demographic groups, and examined co-occurring substance use among U.S. high school students. Public health practitioners, clinicians, school officials, and policymakers can use these findings to expand evidence-based prevention programs, practices, and policies that reduce adolescent substance use risk factors and promote protective factors.

## Methods

### Data Source

This report includes data from the 2009–2021 YRBS, a cross-sectional, school-based survey conducted biennially since 1991. Each survey year, CDC collects data from a nationally representative sample of public and private school students in grades 9–12 in the 50 U.S. states and the District of Columbia. Additional information about YRBS sampling, data collection, response rates, and processing is available in the overview report of this supplement (*8*). The prevalence estimates for current and lifetime alcohol and other substance use for the overall study population and by sex, race and ethnicity, grade, and sexual identity are available at https://nccd.cdc.gov/youthonline/App/Default.aspx. The full YRBS questionnaire, data sets, and documentation are available at https://www.cdc.gov/healthyyouth/data/yrbs/index.htm. This activity was reviewed by CDC and was conducted consistent with applicable federal law and CDC policy.*

### Measures

Four current (i.e., previous 30 days before the survey) and 10 lifetime substance use behaviors were measured. The four current substance use behaviors were alcohol use, marijuana use, binge drinking, and prescription opioid misuse. The 10 lifetime substance use behaviors were alcohol use, marijuana use, inhalant use, ecstasy use, cocaine use, methamphetamine use, heroin use, injection drug use, synthetic marijuana use, and prescription opioid misuse. Use of specific substances was ascertained from questions on frequency of use except for lifetime alcohol use, which was determined from a question on age of initiation. All measures were dichotomized (yes versus no).

Demographic characteristics assessed included sex (female or male), sexual identity (heterosexual; lesbian, gay, or bisexual; or questioning or other), and race and ethnicity (Black or African American [Black], White, and Hispanic or Latino [Hispanic]). (Persons of Hispanic origin might be of any race but are categorized as Hispanic; all racial groups are non-Hispanic.) The numbers of students from other races or multiracial groups were too small for analyses (n<30) for the majority of the substance use measures and were excluded from race and ethnicity analyses. Information on missing data for substance use measures is available in the User’s Guide for each year of data collection at https://www.cdc.gov/healthyyouth/data/yrbs/data.htm.

### Analysis

First, prevalence of each substance use behavior was estimated by survey year during 2009–2021 with available data. Temporal linear and quadratic trends for current and lifetime use of substances were examined using logistic regression models, controlling for sex, grade, and race and ethnicity (https://www.cdc.gov/healthyyouth/data/yrbs/pdf/2019/2019_YRBS_Conducting_Trend_Analyses.pdf). Joinpoint (version 4.9.1.0; National Cancer Institute) was used to identify the year or years where the trend changed direction. Second, 2-year changes in substance use behaviors were assessed by comparing prevalence estimates from 2019 and 2021 using *t*-tests with Taylor series linearization. Changes were considered statistically significant if the p value was <0.05. Third, weighted prevalences of substance use behaviors were estimated for 2019 and 2021 by sex and race and ethnicity. Only 2021 demographic pairwise differences were examined in this report; 2019 estimates by sexual identity and demographic pairwise comparisons were published elsewhere (*2*). Across years, changes in substance use from 2019 to 2021 were assessed using both absolute (i.e., prevalence difference [PD]) and relative (i.e., prevalence ratio [PR]) measures for comparisons by demographic characteristics and frequency of use (https://www.cdc.gov/pcd/issues/2017/16_0516.htm). Changes were considered statistically significant if p values were <0.05 and 95% CIs did not cross zero (for PD) or 1.0 (for PR). Only 2021 data for sexual identity are presented because of a change in the survey question assessing sexual identity from 2019. Finally, prevalences of current co-occurring substance use behaviors (alcohol use, marijuana use, and prescription opioid misuse) among those with any current substance use were calculated. All analyses were conducted using SAS-callable SUDAAN (version 11.0.3; RTI International) to account for the complex sampling design and weighting.

## Results

In 2021, substance use was common among U.S. high school students and varied by substance. Approximately one third of students (30%) reported current use of alcohol or marijuana or prescription opioid misuse. Among current use measures, alcohol (22.7%) and marijuana (15.8%) were the most commonly reported substances used by U.S. high school students (Table 1). Current binge drinking was reported by 10.5% and current prescription opioid misuse by 6.0%. Among lifetime use measures, 47.4% of U.S. high school students reported alcohol use, 27.8% marijuana use, 12.2% prescription opioid misuse, 8.1% inhalant use, and 6.5% synthetic marijuana use. Among lifetime use measures, ecstasy (2.9%), cocaine (2.5%), methamphetamine (1.8%), injection drug use (1.4%), and heroin (1.3%) were less commonly reported.

**TABLE 1:** Trends in prevalence of current and lifetime use of specific substances among high school students — Youth Risk Behavior Survey, United States, 2009–2021*

Behavior/Substance	Prevalence							Linear change^†^	Quadratic change^†^	Change during 2019–2021^§^
	2009 %	2011 %	2013 %	2015 %	2017 %	2019 %	2021 %			
**Current use^¶^**										
Alcohol	41.8	38.7	34.9	32.8	29.8	29.2	22.7	Decreased 2009–2021	No change	Decreased
Marijuana	20.8	23.1	23.4	21.7	19.8	21.7	15.8	Decreased 2009–2021	Increased 2009–2013 Decreased 2013–2021	Decreased
Binge drinking	NA	NA	NA	NA	13.5	13.7	10.5	Decreased 2017–2021	—**	Decreased
Prescription opioid misuse	NA	NA	NA	NA	NA	7.2	6.0	—	—	No change
**Lifetime use**										
Alcohol	68.4	66.7	63.4	60.9	56.5	56.5	47.4	Decreased 2009–2021	Decreased 2009–2017 Decreased 2017–2021	Decreased
Marijuana	36.8	39.9	40.7	38.6	35.6	36.8	27.8	Decreased 2009–2021	Increased 2009–2013 Decreased 2013–2021	Decreased
Inhalants	11.7	11.4	8.9	7.0	6.2	6.4	8.1	Decreased 2009–2021	Decreased 2009–2017 Increased 2017–2021	Increased
Ecstasy	6.7	8.2	6.6	5.0	4.0	3.6	2.9	Decreased 2009–2021	No change 2009–2013 Decreased 2013–2021	No change
Cocaine	6.4	6.8	5.5	5.2	4.8	3.9	2.5	Decreased 2009–2021	No change 2009–2017 Decreased 2017–2021	Decreased
Methamphetamine	4.1	3.8	3.2	3.0	2.5	2.1	1.8	Decreased 2009–2021	No change	No change
Heroin	2.5	2.9	2.2	2.1	1.7	1.8	1.3	Decreased 2009–2021	No change	No change
Injection drug use	2.1	2.3	1.7	1.8	1.5	1.6	1.4	Decreased 2009–2021	No change	No change
Synthetic marijuana	NA	NA	NA	9.2	6.9	7.3	6.5	Decreased 2015–2021	—	No change
Prescription opioid misuse	NA	NA	NA	NA	14.0	14.3	12.2	Decreased 2017–2021	—	Decreased

**Abbreviation**: NA = not available.

* 2009: N = 16,410 respondents; 2011: N = 15,425 respondents; 2013: N = 13,583 respondents; 2015: N = 15,624 respondents; 2019: N = 13,677 respondents; 2021: N = 17,232 respondents. Because the state and local questionnaires differ by jurisdiction, students in these schools were not asked all national YRBS questions. Therefore, the total number (N) of students answering each question varied. Percentages in each category are calculated on the known data.

^†^ On the basis of trend analyses from a logistic regression model controlling for sex, race and ethnicity, and grade (p<0.05).

^§^ On the basis of *t*-test analysis with Taylor series linearization (p<0.05).

^¶^ Previous 30 days before the survey.

** Dashes indicate insufficient years of data to assess trends.

Trend data were available for all substance use measures except current prescription opioid misuse. All substance use measures with available trend data decreased linearly over the period assessed (2009–2021 for most substances, 2015–2021 for lifetime synthetic marijuana use, and 2017–2021 for current binge drinking and lifetime prescription opioid misuse). From 2019 to 2021, prevalence of current substance use decreased for alcohol (from 29.2% to 22.7%), marijuana (from 21.7% to 15.8%), and binge drinking (from 13.7% to 10.5%). No change was observed in prevalence of current prescription opioid misuse. Lifetime alcohol use, marijuana use, cocaine use, and prescription opioid misuse also decreased from 2019 to 2021; lifetime inhalant use increased from 6.4% to 8.1%.

Compared with males, females had a higher prevalence of current substance use in 2021 for alcohol (26.8% versus 18.8%), marijuana (17.8% versus 13.6%), binge drinking (12.2% versus 9.0%), and prescription opioid misuse (8.0% versus 4.0%) (Table 2). Females also had a higher prevalence of lifetime alcohol use (53.2% versus 42.0%), lifetime marijuana use (30.9% versus 24.8%), lifetime prescription opioid misuse (14.8% versus 9.5%), and lifetime inhalant use (9.4% versus 6.8%) compared with males. However, males had a higher prevalence of lifetime heroin use (1.6% versus 0.8%) and injection drug use (1.7% versus 0.9%).

**TABLE 2:** Prevalence of and changes in prevalence of current and lifetime use of specific substances among high school students, by sex — Youth Risk Behavior Survey, United States, 2019 and 2021*

Behavior/Substance	Sex							
	Male				Female			
	2019 %	2021 %	PD (95% CI)	PR (95% CI)	2019 %	2021 %	PD (95% CI)	PR (95% CI)
**Current use^†^**								
Alcohol	26.4	18.8	−7.7 (−0.3 to −5.1)^§^	0.7 (0.6 to 0.8)^§^	31.9	26.8^¶^	−5.1 (−8.3 to −1.9)^§^	0.8 (0.8 to 0.9)^§^
Marijuana	22.5	13.6	−8.9 (−1.3 to −6.4)^§^	0.6 (0.5 to 0.7)^§^	20.8	17.8^¶^	−3.0 (−6.0 to 0.0)	0.9 (0.7 to 1.0)
Binge drinking	12.7	9.0	−3.7 (−5.6 to −1.7)^§^	0.7 (0.6 to 0.8)^§^	14.6	12.2^¶^	−2.5 (−5.2 to 0.2)	0.8 (0.7 to 1.0)
Prescription opioid misuse	6.1	4.0	−2.1 (−3.5 to −0.8)^§^	0.7 (0.5 to 0.9)^§^	8.3	8.0^¶^	−0.3 (−2.2 to 1.6)	1.0 (0.8 to 1.2)
**Lifetime use**								
Alcohol	53.1	42.0	−11.1 (−14.2 to −8.0)^§^	0.8 (0.7 to 0.8)^§^	60.0	53.2^¶^	−6.9 (−10.2 to −3.5)^§^	0.9 (0.8 to 0.9)^§^
Marijuana	37.0	24.8	−12.3 (−15.9 to −8.7)^§^	0.7 (0.6 to 0.8)^§^	36.5	30.9^¶^	−5.6 (−9.3 to −1.9)^§^	0.9 (0.8 to 1.0)^§^
Inhalants	5.7	6.8	1.1 (−0.1 to 2.3)	1.2 (1.0 to 1.5)	6.9	9.4^¶^	2.5 (1.1 to 3.9)^§^	1.4 (1.1 to 1.6)^§^
Ecstasy	4.6	2.9	−1.7 (−2.8 to −0.7)^§^	0.6 (0.5 to 0.8)^§^	2.4	2.7	0.4 (−0.5 to 1.3)	1.2 (0.8 to 1.7)
Cocaine	4.9	2.6	−2.3 (−3.3 to −1.4)^§^	0.5 (0.4 to 0.7)^§^	2.7	2.2	−0.5 (−1.6 to 0.5)	0.8 (0.5 to 1.2)
Methamphetamine	2.7	1.9	−0.8 (−1.6 to 0.0)	0.7 (0.5 to 1.0)	1.5	1.4	−0.1 (−0.8 to 0.6)	1.0 (0.6 to 1.5)
Heroin	2.3	1.6	−0.7 (−1.5 to 0.1)	0.7 (0.5 to 1.0)	1.0	0.8^¶^	−0.3 (−0.9 to 0.4)	0.8 (0.4 to 1.5)
Injection drug use	2.1	1.7	−0.4 (−1.2 to 0.4)	0.8 (0.5 to 1.2)	1.1	0.9^¶^	−0.2 (−0.9 to 0.6)	0.9 (0.4 to 1.8)
Synthetic marijuana	7.2	5.8	−1.4 (−2.9 to 0.1)	0.8 (0.6 to 1.0)	7.4	7.1	−0.3 (−1.9 to 1.3)	1.0 (0.8 to 1.2)
Prescription opioid misuse	12.4	9.5	−2.9 (−4.7 to −1.2)^§^	0.8 (0.7 to 0.9)^§^	16.1	14.8^¶^	−1.4 (−3.9 to 1.1)	0.9 (0.8 to 1.1)

**Abbreviations:** PD = prevalence difference; PR = prevalence ratio.

* 2019: N = 13,677 respondents; 2021: N = 17,232 respondents. Because the state and local questionnaires differ by jurisdiction, students in these schools were not asked all national YRBS questions. Therefore, the total number (N) of students answering each question varied. Percentages in each category are calculated on the known data.

^†^ Previous 30 days before the survey.

^§^ Statistically significant results (p<0.05).

^¶^ Significantly different from male students in 2021, on the basis of *t*-test analysis with Taylor series linearization (p<0.05).

Changes in substance use from 2019 to 2021 varied by sex (Table 2). Current alcohol use decreased for both females and males. Males also had a 3.7% absolute decrease and a 30% relative decrease in binge drinking and a 2.1% absolute decrease and a 30% relative decrease in current prescription opioid misuse. Among lifetime use measures, alcohol and marijuana use decreased among both females and males. Decreases also were observed in ecstasy use, cocaine use, and prescription opioid misuse for males. However, for females, a 2.5% absolute increase and a 40% relative increase occurred in inhalant use from 2019 to 2021.

Prevalence of substance use measures varied by racial and ethnic group, with different groups reporting higher prevalences of use for different substances. For example, Black students reported a higher prevalence of current marijuana use (20.5%) compared with Hispanic (16.7%) and White (14.8%) students (Table 3). Black students reported a lower prevalence of current alcohol use (13.2%) compared with White (25.9%) and Hispanic (22.9%) students. White students reported a lower prevalence of current prescription opioid misuse (4.6%) compared with Black (8.6%) and Hispanic (8.3%) students.

**TABLE 3:** Prevalence of and changes in prevalence of current and lifetime use of specific substances among high school students, by race and ethnicity — Youth Risk Behavior Survey, United States, 2019 and 2021*

Behavior/Substance	Race and ethnicity^†^											
	Black or African American				White				Hispanic or Latino			
	2019 %	2021 %	PD (95% CI)	PR (95% CI)	2019 %	2021 %	PD (95% CI)	PR (95% CI)	2019 %	2021 %	PD (95% CI)	PR (95% CI)
**Current use^§^**												
Alcohol	16.8	13.2^¶^	−3.6 (−7.7 to 0.5)	0.8 (0.6 to 1.0)	34.2	25.9	−8.3 (−11.4 to −5.3)**	0.8 (0.7 to 0.8)**	28.4	22.9^¶,††^	−5.5 (−9.5 to −1.6)**	0.8 (0.7 to 1.0)**
Marijuana	21.7	20.5^¶^	−1.2 (−5.4 to 2.9)	0.9 (0.8 to 1.2)	22.1	14.8	−7.3 (−10.2 to −4.5)**	0.7 (0.6 to 0.8)**	22.4	16.7^††^	-5.7 (−9.2 to −2.2)**	0.7 (0.6 to 0.9)**
Binge drinking	6.2	4.1^¶^	−2.2 (−4.8 to 0.5)	0.7 (0.4 to 1.0)	17.3	13.3	−4.0 (−6.6 to −1.4)**	0.8 (0.7 to 0.9)**	12.4	10.1^¶,††^	−2.3 (−4.8 to 0.1)	0.8 (0.7 to 1.0)
Prescription opioid misuse	8.7	8.6^¶^	−0.1 (−4.2 to 3.9)	1.0 (0.6 to 1.6)	5.5	4.6	−1.0 (−2.5 to 0.5)	0.8 (0.6 to 1.1)	9.8	8.3^¶^	−1.5 (−4.0 to 1.1)	0.9 (0.6 to 1.1)
**Lifetime use**												
Alcohol	47.2	39.4^¶^	−7.8 (−13.5 to −2.0)**	0.8 (0.7 to 1.0)**	58.8	50.0	−8.8 (−12.0 to −5.6)**	0.9 (0.8 to 0.9)**	60.4	50.4^††^	−10.0 (−14.5 to −5.5)**	0.8 (0.8 to 0.9)**
Marijuana	37.5	33.3^¶^	−4.2 (−10.5 to 2.2)	0.9 (0.7 to 1.1)	36.8	26.2	−10.7 (−14.1 to −7.2)**	0.7 (0.6 to 0.8)**	39.2	31.2^¶^	−7.9 (−12.5 to −3.4)**	0.8 (0.7 to 0.9)**
Inhalants	7.2	7.0	−0.2 (−2.5 to 2.1)	1.0 (0.7 to 1.3)	6.3	8.3	1.9 (0.3 to 3.6)**	1.3 (1.1 to 1.6)**	6.6	8.2	1.6 (−0.1 to 3.3)	1.2 (1.0 to 1.6)
Ecstasy	3.8	2.7	−1.1 (−2.9 to 0.7)	0.7 (0.4 to 1.2)	2.7	2.9	0.1 (−0.9 to 1.2)	1.1 (0.7 to 1.5)	4.4	2.7	-1.7 (−2.7 to −0.7)**	0.6 (0.5 to 0.8)**
Cocaine	4.0	1.9	−2.1 (−3.8 to −0.4)**	0.5 (0.3 to 0.8)**	2.9	2.4	−0.5 (−1.4 to 0.4)	0.8 (0.6 to 1.1)	5.6	2.9	−2.7 (−4.4 to −1.0)**	0.5 (0.3 to 0.8)**
Methamphetamine	3.8	2.0	−1.9 (−3.7 to 0.0)	0.5 (0.3 to 0.9)**	1.2	1.4	0.2 (−0.3 to 0.7)	1.2 (0.8 to 1.7)	2.7	2.3^¶^	−0.4 (−1.6 to 0.8)	0.9 (0.5 to 1.3)
Heroin	3.4	1.7	−1.7 (−3.4 to −0.1)**	0.5 (0.3 to 0.9)**	0.9	1.0	0.1 (−0.3 to 0.5)	1.2 (0.8 to 1.8)	2.4	1.6^¶^	−0.9 (−2.1 to 0.4)	0.7 (0.4 to 1.1)
Injection drug use	2.9	1.9	−0.9 (−3.0 to 1.1)	0.7 (0.3 to 1.5)	0.8	1.1	0.3 (−0.3 to 0.8)	1.4 (0.8 to 2.4)	2.5	1.8	−0.7 (−1.8 to 0.3)	0.7 (0.4 to 1.2)
Synthetic marijuana	5.7	6.8	1.1 (−1.2 to 3.3)	1.2 (0.8 to 1.7)	6.7	6.5	−0.2 (−1.6 to 1.3)	1.0 (0.8 to 1.2)	9.8	6.8	−3.1 (−4.9 to −1.3)**	0.7 (0.6 to 0.9)**
Prescription opioid misuse	15.3	13.6	−1.7 (−5.4 to 1.9)	0.9 (0.7 to 1.1)	12.7	11.2	−1.4 (−3.7 to 0.8)	0.9 (0.7 to 1.1)	16.0	13.8	−2.2 (−5.5 to 1.2)	0.9 (0.7 to 1.1)

**Abbreviations:** PD = prevalence difference; PR = prevalence ratio.

* 2019: N = 13,677 respondents; 2021: N = 17,232 respondents. Because the state and local questionnaires differ by jurisdiction, students in these schools were not asked all national YRBS questions. Therefore, the total number (N) of students answering each question varied. Percentages in each category are calculated on the known data.

^†^ Persons of Hispanic or Latino (Hispanic) origin might be of any race but are categorized as Hispanic; all racial groups are non-Hispanic.

^§^ Previous 30 days before the survey.

^¶^ Significantly different from White students in 2021, on the basis of *t*-test analysis with Taylor series linearization (p<0.05).

** Statistically significant results (p<0.05).

^††^ Significantly different from Black or African American students in 2021, on the basis of *t*-test analysis with Taylor series linearization (p<0.05).

By race and ethnicity, current and lifetime marijuana use decreased for both White and Hispanic high school students, and lifetime alcohol use decreased for all three racial and ethnic groups from 2019 to 2021. White students reported less binge drinking in 2021 compared with 2019 and more lifetime inhalant use. Hispanic students reported decreases in lifetime ecstasy use, cocaine use, and synthetic marijuana use. Lifetime use measures for cocaine, methamphetamine, and heroin decreased among Black students.

Prevalence of all substance use measures varied by sexual identity in 2021, with students identifying as lesbian, gay, or bisexual reporting a higher prevalence of all current and lifetime substance use measures compared with students identifying as heterosexual (Table 4). Compared with students who identified as heterosexual, students who identified as questioning or other reported a higher prevalence of current marijuana use and prescription opioid misuse, and a higher prevalence of all lifetime use measures except for lifetime alcohol use, marijuana use, and synthetic marijuana use. However, compared with students who identified as lesbian, gay, or bisexual, students who identified as questioning or other reported a lower prevalence of most current use measures (alcohol use, marijuana use, and binge drinking) and multiple lifetime use measures (alcohol, marijuana, ecstasy, and synthetic marijuana). Frequency of current and lifetime use among high school students reporting use of specific substances in 2021 was not substantially different from 2019 (Supplementary Table, https://stacks.cdc.gov/view/cdc/125216) (*2*).

**TABLE 4:** Prevalence of current and lifetime use of specific substances among high school students, by sexual identity — Youth Risk Behavior Survey, United States, 2021*

Behavior/Substance	Heterosexual %	Lesbian, gay, or bisexual %	Questioning or other %
**Current use^†^**			
Alcohol	21.6	29.3^§^	20.9^¶^
Marijuana	14.0	25.6^§^	16.5^§,¶^
Binge drinking	10.3	13.6^§^	7.6^§,¶^
Prescription opioid misuse	4.3	11.7^§^	10.3^§^
**Lifetime use**			
Alcohol	45.8	58.0^§^	46.2^¶^
Marijuana	25.8	41.2^§^	27.5^¶^
Inhalants	6.0	15.1^§^	13.4^§^
Ecstasy	2.1	6.0^§^	3.9^§,¶^
Cocaine	1.8	4.4^§^	3.1^§^
Methamphetamine	1.1	3.4^§^	3.0^§^
Heroin	0.8	1.9^§^	2.4^§^
Injection drug use	1.0	1.9^§^	2.7^§^
Synthetic marijuana	5.9	9.7^§^	6.1^¶^
Prescription opioid misuse	9.4	21.5^§^	18.6^§^

* N = 17,232 respondents. Because the state and local questionnaires differ by jurisdiction, students in these schools were not asked all national YRBS questions. Therefore, the total number (N) of students answering each question varied. Percentages in each category are calculated on the known data.

^†^ Previous 30 days before the survey.

^§^ Significantly different from heterosexual students, based on *t*-test analysis with Taylor series linearization (p<0.05).

^¶^ Significantly different from lesbian, gay, or bisexual students, based on *t*-test analysis with Taylor series linearization (p<0.05).

Students commonly reported current co-occurring substance use (Figure). Among high school students who reported current alcohol use, marijuana use, or prescription opioid misuse, 35.1% reported using two or more substances. Alcohol and marijuana were the most commonly co-used substances among those who reported any current substance use, with 30.2% reporting co-use. Alcohol use and prescription opioid misuse was reported by 7.9%, marijuana use and prescription opioid misuse by 6.7%, and use (misuse) of all three substances by 4.8%.

**FIGURE:**
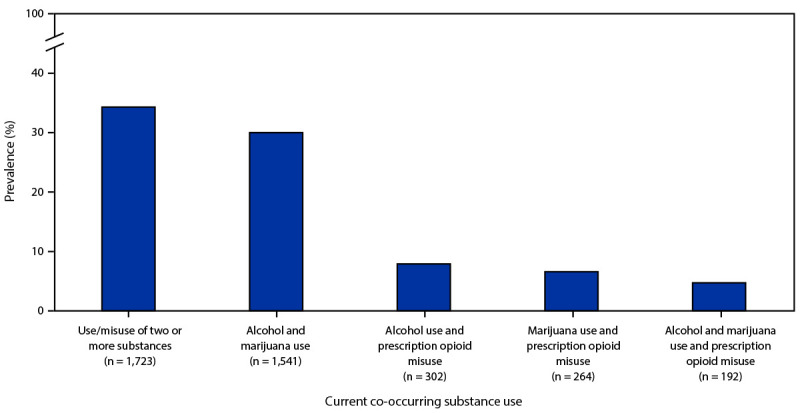
Prevalence of current* co-occurring substance use among high school students who reported any current substance use^†^ — Youth Risk Behavior Survey, United States, 2021 * Previous 30 days before the survey; n = 5,203 high school students who reported any current substance use. This n represents students who reported current use of at least one of the three substances, regardless of potential missing values for the other two substances. ^†^ Current substance use measures were current alcohol use, current marijuana use, and current prescription opioid misuse. Missing observations were excluded in the calculation of percentages in each category. For alcohol and marijuana use, missing = 11 of 5,023 (0.2%); for marijuana use and prescription opioid misuse, missing = 37 of 5,023 (0.7%); for alcohol use and prescription opioid misuse, missing = 56 of 5,023 (1.1%).

## Discussion

This report documents that substance use prevalence among U.S. high school students had been declining for a decade before the COVID-19 pandemic. For the majority of substance use outcomes, prevalence further declined from 2019 to 2021, including for current alcohol use, marijuana use, and binge drinking and for lifetime alcohol use, marijuana use, cocaine use, and prescription opioid misuse. Despite these declines, approximately one in three high school students (30%) reported past 30-day substance use in 2021. Among those reporting current substance use, approximately 35% used two or more substances, suggesting that use of multiple substances is common, an important consideration when implementing prevention and intervention strategies. The decline in adolescent substance use during the COVID-19 pandemic is consistent with other studies of U.S. adolescents, including from the Monitoring the Future study, which also reported significant decreases in lifetime and past 30-day marijuana use, binge drinking, and lifetime cocaine and heroin use in 2021 (*1*).

This report highlights disparities in substance use by race and ethnicity and sexual identity. For example, current and lifetime marijuana use decreased from 2019 to 2021 among White and Hispanic students, whereas no change was noted for Black students. These disparities could be the result of exacerbation of preexisting health inequities, such as access to prevention and treatment services, experiences of racism and historical trauma, and economic challenges (*9*). Social determinants of health specific to COVID-19, such as disproportionate representation of parents and caregivers among front line workers and being in a family that experienced COVID-19–related severe illness and death, might also have influenced outcome disparities (*9*,*10*). In addition, the higher prevalence estimates of current and lifetime substance use in 2021 among students identifying as lesbian, gay, or bisexual compared with students identifying as heterosexual are generally consistent with the results from the 2019 YRBS (*2*). This finding could be a result of increased experiences of violence and other types of victimization, discrimination, adversity, and isolation that these adolescents might have experienced (*11*).

An analysis of National Survey on Drug Use and Health data found that among adolescents and adults who reported drinking alcohol and misusing prescription pain relievers, approximately 40% misused a prescription pain reliever while drinking or within a couple of hours of drinking alcohol (*12*). In this context, the finding of high rates of using two or more substances among U.S. high school students who reported substance use is particularly concerning. Using alcohol and other substances increases the risk for health problems and overdose and can increase the effects of the substances if the substances are used at the same time (https://www.cdc.gov/alcohol/fact-sheets/alcohol-and-other-substance-use.html). In addition, although the prevalence of current prescription opioid misuse did not change among high school students, adolescent overdose deaths have increased substantially in recent years (*4*), in parallel to increased availability of counterfeit pills containing illicitly made fentanyl (https://www.dea.gov/press-releases/2021/05/21/dea-issues-warning-over-counterfeit-pills). That finding suggests an urgent need for new strategies to raise awareness among adolescents about exposure to highly lethal substances disguised as commonly misused prescription drugs and for expanded access to harm reduction interventions such as naloxone and fentanyl test strips.

The declines in adolescent substance use might be partially explained by pandemic-specific contextual factors, including decreased access to substances because of reduced contact with peers and increases in parental supervision (*13*). Inhalant use increased, a finding consistent with other research, and might also be the result of access. Inhalants (i.e., noncombusted and nonheated gases that can be inhaled for euphoric effect) are easily accessible inside most homes (*1*). Consequently, it is possible that as social interactions resume, access to substances could increase, supervision might decrease, and adolescent substance use could revert to prepandemic levels (*1*).

Effective strategies to prevent and mitigate adolescent substance use are multilevel and focus on reducing risk factors associated with use and increasing protective factors likely to decrease use in the environments where adolescents interact (https://addiction.surgeongeneral.gov/sites/default/files/surgeon-generals-report.pdf). Feeling connected to family, positive peers (those not engaging in substance use risk behaviors), school, and community is an important protective factor that can buffer against adverse childhood experiences (ACEs), poor mental health, and health risk behaviors, including substance use and sexual risk behaviors (*14*). Family and parent substance use programs that focus on parental communication, monitoring, and modeling of positive problem-solving and coping strategies, can be effective in influencing adolescents’ substance use behavior (*14*). Interventions that promote a positive school climate and increase students’ feelings of connectedness to the school and decrease student dissatisfaction, in conjunction with effective health education, can improve substance use outcomes (*15*). For example, CDC’s What Works in Schools approach (https://www.cdc.gov/healthyyouth/whatworks/index.htm), focused on creating safe and supportive environments, effective health education, and linking teens to health services, has demonstrated an effect on various mental health and health outcomes, including substance use.

Community–school partnerships that increase access to evidence-based substance use prevention curricula and substance use treatment services also have demonstrated protective effects on substance use into adulthood for both illicit drugs and prescription drug misuse, such as PROmoting School-community-university Partnerships to Enhanced Resilience (PROSPER) and Communities That Care (CTC) (https://store.samhsa.gov/sites/default/files/d7/priv/pep19-pl-guide-1.pdf). The majority of adolescents are registered in school; therefore, schools can have an important role in substance use prevention and treatment by providing a supportive school environment including access to a counselor or a psychologist; school policies regarding the use of tobacco products, alcohol, and marijuana; and evidence-based programs to prevent substance use and violence and promote coping and problem-solving skills and mental health (*16*).

Youth substance use can also be reduced and prevented with evidence-based policies that reduce the availability of substances where youths live and decrease their access to them (https://addiction.surgeongeneral.gov/sites/default/files/surgeon-generals-report.pdf). One example is to reduce the number and concentration of places that sell alcohol. Increasing the price of alcohol through alcohol taxes, enhanced enforcement of laws that prohibit sales of marijuana and alcohol to minors, and enforcement of other substance use policies (e.g., prescription drug monitoring programs) also can reduce adolescent substance use (https://www.cdc.gov/alcohol/fact-sheets/alcohol-and-other-substance-use.html; https://www.thecommunityguide.org/topics/excessive-alcohol-consumption.html).

Disparities occur in adolescent substance use by race and ethnicity as well as sexual identity. Tailoring adolescent substance use prevention strategies to reach different population subgroups can be effective when implemented in tandem with broader strategies that prevent and mitigate ACEs and other individual, family, school, and community factors that influence risk for substance use (https://www.cdc.gov/violenceprevention/pdf/preventingACES.pdf).

## Limitations

General limitations for the YRBS are available in the overview report of this supplement (*8*). The findings in this report are subject to at least three additional limitations. First, the survey questions on prescription opioid misuse refer to prescription pain medications and then provide examples of medications containing opioids only. Prescription opioid misuse prevalence might be overestimated if respondents included the use of nonopioid prescription pain medications; however, overestimation of prevalence should not have affected measures of difference between survey years. Second, substantial data were missing for certain substance use variables (e.g., prescription opioid misuse), which might be because of the order of the survey questions or other factors related to survey administration (*2*). These missing data could have resulted in overestimation or underestimation of prevalence. Finally, the YRBS questionnaire was updated in 2021 to be more inclusive of student sexual identities. This change limited the ability to assess changes in substance use by sexual identity in 2021 compared with earlier years.

## Conclusion

Youth substance use has declined over the past decade, including during the COVID-19 pandemic; however, substance use remains common among U.S. high school students, and continued monitoring is important in the context of the changing marketplaces for alcohol beverage products and other drugs. Scaling-up tailored, evidence-based policies, programs, and practices to reduce factors that contribute to risk for adolescent substance use and promote factors that protect against risk might help build on recent declines.

## Footnotes

* See e.g., 45 C.F.R. part 46.102(l)(2), 21 C.F.R. part 56; 42 U.S.C. §241(d); 5 U.S.C. §552a; 44 U.S.C. §3501 et seq.
