# *Erratum:* Vol. 72, No. Suppl-1

**DOI:** 10.15585/mmwr.mm7227a5

**Published:** 2023-07-07

In the Supplement “Youth Risk Behavior Surveillance — United States, 2021,” several errors occurred in the report “Alcohol and Other Substance Use Before and During the COVID-19 Pandemic Among High School Students — Youth Risk Behavior Survey, United States, 2021.”

On page 84, in line 13 of the Abstract, the percentage should have read, “(**30%**),” and in line 14, the percentage should have read, “**35%**.”

On page 87, the first sentence of the first footnote under Table 1 should have read, “* 2009: N = 16,410 respondents; 2011: N = 15,425 respondents; 2013: N = 13,583 respondents; 2015: N = 15,624 respondents; **2017: N = 14,765 respondents;** 2019: N = 13,677 respondents; 2021: N = 17,232 respondents.”

On page 91, the first sentence of the first footnote under the Figure should have read, “* Previous 30 days before the survey; **n = 5,023** high school students who reported any current substance use.”

